# Supplementary material for: Flexible integration of free-standing nanowires into silicon photonics
Source: Nat Commun. 2017 Jun 14;8:20. doi: 10.1038/s41467-017-00038-0 (PMC5471269; doi:10.1038/s41467-017-00038-0)
Supplement: Supplementary file 1 — Supplementary Information [file 41467_2017_38_MOESM1_ESM.pdf]

File name: Supplementary Information
Description: Supplementary Figures, Supplementary Notes and Supplementary References

File name: Peer Review File
Description:

Supplementary Information

Supplementary Note 1. Morphology characterization of CdS freestanding nanowires (FNWs)

The morphology of CdS FNWs was examined by scanning electron microscopy (SEM), as depicted in Supplementary Figs. 1a and 1b. Supplementary Fig. 1a shows typical as-grown CdS FNWs on a silicon substrate. Supplementary Fig. 1b shows a single 690-nm-diameter CdS FNW transferred onto a clean silicon wafer, with highly uniform geometrical shape and smooth surface. For better resolution, Supplementary Fig. 1c shows a transmission electron microscopy (TEM) image of a sidewall area on the CdS FNW, from which we estimated an RMS roughness of about 0.3 nm. High resolution TEM image in Supplementary Fig. 1d demonstrates clear lattice fringes, revealing the excellent crystal structure of the FNW. Measured interlayer spacing of about 0.32 nm indicates the (101) planes of bulk hexagonal CdS[1]. Corresponding selected area electron diffraction (SAED) pattern is depicted in the inset of Supplementary Fig. 1d, confirming the single crystalline of the FNW.

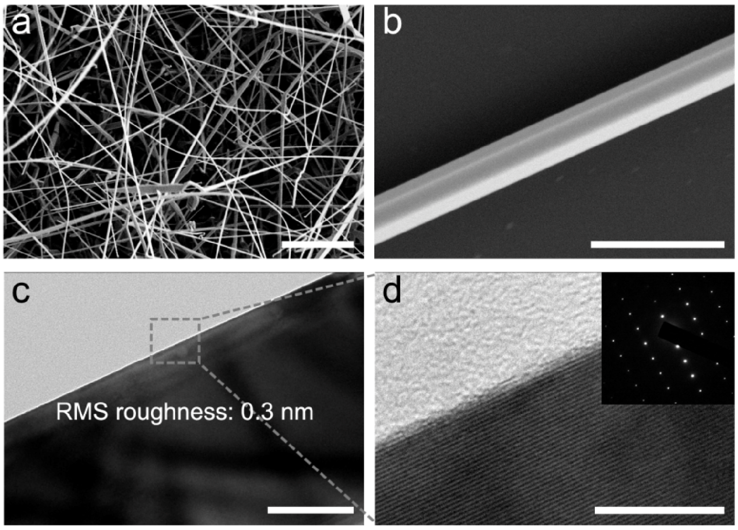

Supplementary Figure 1. a, SEM image of as-grown CdS FNWs on a silicon substrate. Scale bar, 10 μm. **b**, SEM image of a 690-nm-diameter CdS FNW. Scale bar, 2 μm. **c**, TEM image of a sidewall area on a CdS FNW. Scale bar, 100 nm. **d**, High resolution TEM image of the dashed frame area in **c**. Inset gives the corresponding SAED pattern. Scale bar, 10 nm.

Supplementary Note 2. Micromanipulation of FNWs for integration with silicon waveguides

CdS FNWs were first dispersed in ethanol, and then deposited onto a glass slide. After the FNWs were dried in the open air, they can be manipulated by tapered fibre probes mounted on 3-dimension moving stages under an optical microscope[2]. Supplementary Fig. 2a gives an SEM image of a typical tapered fibre probe drawn from a single mode fibre (Corning SMF-28), with tip size down to 50 nm. To pick up a CdS FNW, first we used a fibre probe to insert beneath one end of the FNW and lift it up slowly, while the rest part of the FNW kept attached on the glass slide due to the strong friction force between the FNW and the slide (Supplementary Figs. 2c and 2d). Then we used another fibre probe to lift up the other end of the FNW with the same approach, and finally suspended the FNW across the two fibre probe in the air (Supplementary Fig. 2e). Generally, longer and thicker FNWs are easier to be manipulated. However, using the nanoscale

fibre tip shown in Supplementary Fig. 2a, we are also able to manipulate tiny FNWs with a length down to 8 μm [Ref. 3] and/or a diameter down to 100 nm [Ref. 4].

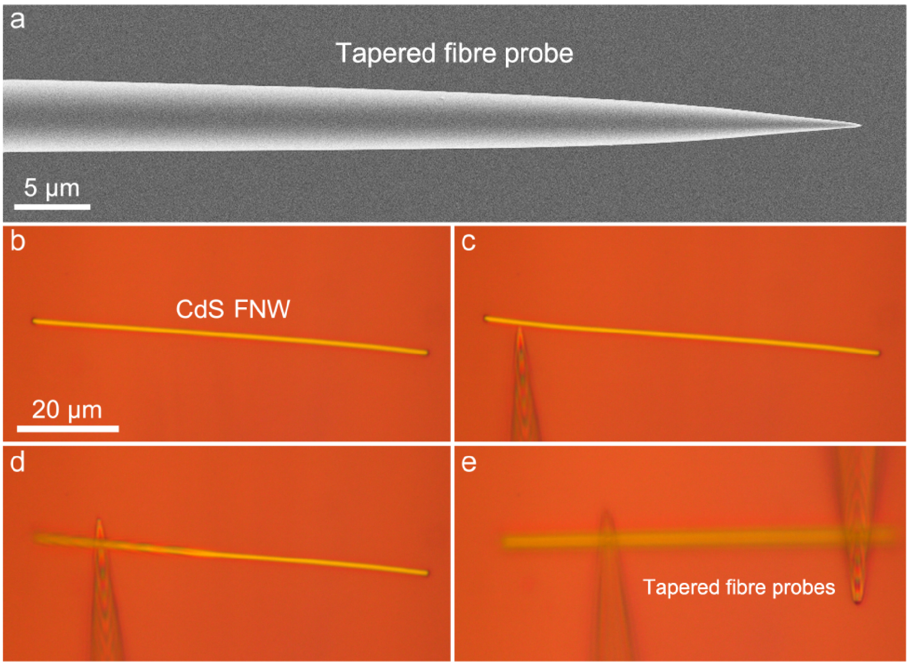

Supplementary Figure 2. **a**, SEM image of a tapered fibre probe. **b**, Optical micrograph of a CdS FNW on a glass slide. **c** and **d**, Pick up one end of the FNW with a fibre probe. **e**, Pick up the other end of the FNW with another fibre probe and suspend the FNW in the air. Scale bar in **b** applies to **c**-**e**.

After replacing the glass slide by a silicon chip, we roughly aligned a target silicon waveguide (SW) circuit on the chip to the suspended CdS FNW under the optical microscope for subsequent FNW assembling. Supplementary Fig. 3 shows the procedure of bridging two SWs with a CdS FNW in a side-by-side coupling scheme. First, the FNW was transferred to a place close to the SWs and dropped down onto the SiO_2 substrate (Supplementary Figs. 3a and 3b). Then we used a fibre probe to press on the FNW to make it totally attached on the substrate (Supplementary Fig. 3c). Finally, the FNW was pushed carefully to contact with the SW ends (Supplementary Figs. 3d and 3e). Being totally attached on a clean substrate, FNWs are usually stable enough for

afterward top-down processing, which suggests that electrically active FNWs can also be used for this FNW-SW integration by adding electrical leads.

For the side-coupling scheme, the nanowire was supported by the SiO_2 substrate instead of the silicon core, as shown in Fig. 1 and Fig. 2 in the main text. In this case, when assembling the nanowire-waveguide coupling devices, we placed a nanowire on the SiO_2 substrate and then pushed it towards a target silicon waveguide (Supplementary Fig. 3) until it was stopped by the silicon core. We double-checked the side-coupling structure by using a top-viewed SEM image depicted in the lower panel of Fig. 1b. From this figure, one sees the nanowire and silicon waveguide simultaneously, which means that the nanowire is at the ***side*** of the silicon core. In contrast, for the vertical coupling case, a part of the silicon waveguide can not be seen because the nanowire on the ***top*** is larger than the silicon core, as shown in Figs. 3b-3c and 4a.

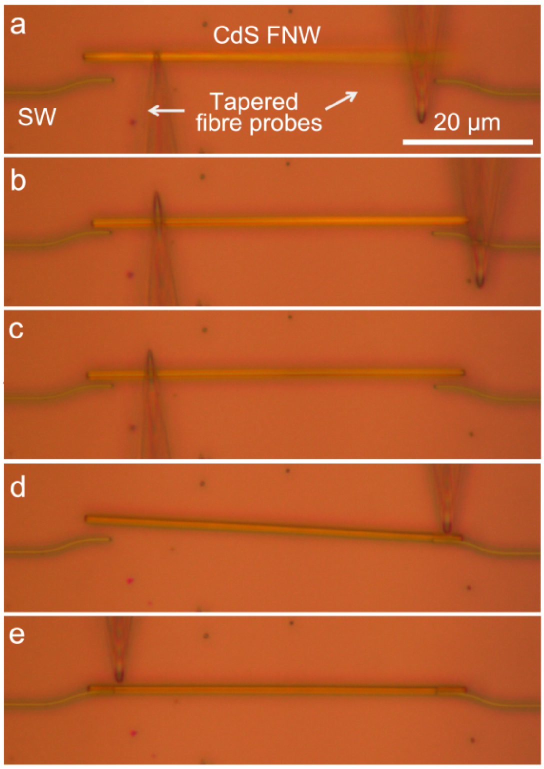

Supplementary Figure 3. Procedure of bridging two SWs with a CdS FNW using a side-by-side coupling scheme. **a** and **b**, Transfer and drop down the FNW onto the SiO_2 substrate near the SW pair. **c**, Press on the FNW by a fibre probe to make it totally attached on the substrate. **d** and **e**, Push the FNW to contact with the SW ends. Scale bar in **a** applies to **b**-**e**.

Supplementary Note 3. Effective indices of CdS FNWs and SWs

Supplementary Fig. 4 gives the calculated effective index (EI) dispersions of fundamental quasi-TE modes in SWs (dashed lines) and SiO_2-supported CdS FNWs with different diameters (solid lines), respectively. The height of the SWs is 340 nm. In the calculation, the refractive indices of bulk silicon and CdS used at telecommunication band are about 3.48 [Ref. 5] and 2.26 [Ref. 6], respectively. From 1,520 to 1,620 nm, we can see the EI dispersion curves of the 290 and 300-nm-width SWs have intersection points with that of the FNWs (circles in Supplementary Fig. 4), where the phase matching between CdS FNWs and SWs are realized. For the 860-nm-diameter

CdS FNW used in our experiment, the EI difference with the 320-nm-width SW decreases with increasing wavelength, while the case for the 280-nm-width SW shows an opposite behavior. These results can qualitatively explain the wavelength-dependent behavior of coupling efficiency in Fig. 1d. In contrast, EI of the 300-nm-width SW is much closer to that of the 860-nm-diameter FNW, resulting in higher average coupling efficiency in Fig. 1d.

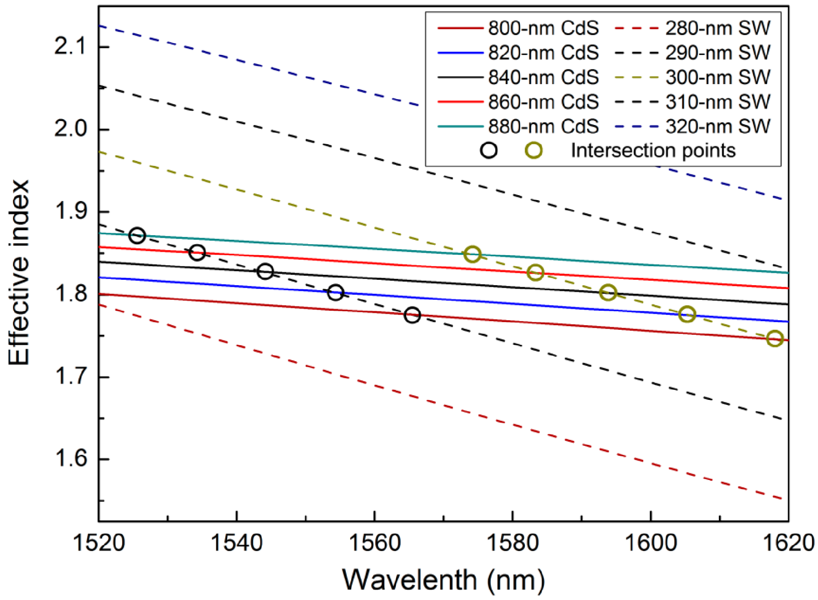

Supplementary Figure 4. EI dispersions of fundamental quasi-TE modes in SWs and CdS FNWs calculated by COMSOL. Solid lines indicate the dispersion curves of CdS FNWs with diameters of 800-880 nm, respectively. Dashed lines indicate the dispersion curves of SWs with widths of 280-320 nm, respectively.

Supplementary Note 4. Simulation of the near-field optical coupling between a CdS FNW and an SW

We used Lumerical FDTD to simulate the near-field optical coupling between an SW and a CdS FNW. Supplementary Fig. 5a shows the schematic illustration of the side-by-side coupling scheme with five dashed cross-section planes for investigation (Planes 1-5). The coupling length is 2.9 μm

and the wavelength is 1,580 nm. From the power density distributions on Plane 1 to Plane 5 (corresponding to Supplementary Figs. 5b-5f, respectively), we can clearly see that the optical energy gradually transfers from the SW to the FNW within the coupling area during its propagating. Power density distributions on horizontal xz planes across the central axes of the SW and the FNW are depicted in Supplementary Figs. 5g and 5h, respectively, clearly demonstrating the flowing of optical energy from the SW to the CdS FNW.

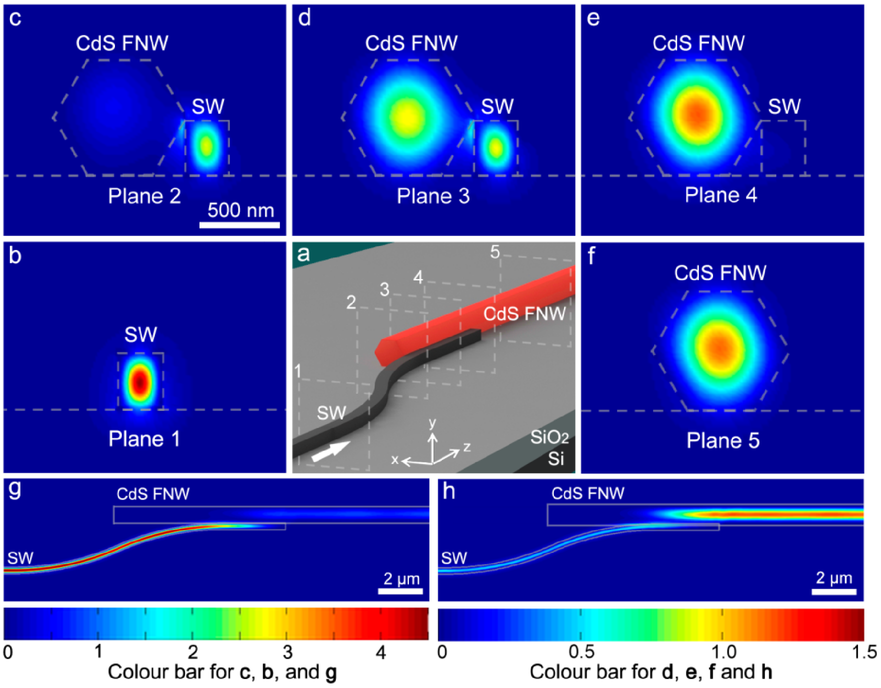

Supplementary Figure 5. **a**, Side-by-side FNW-SW coupling scheme diagram. The white arrow indicates the direction of light propagation. The FNW diameter and the SW width are 860 nm and 290 nm, respectively. **b**-**f**, power density distributions on Planes 1-5 indicated in **a**. Scale bar in **c** applies to **b** and **d**-**f**. **g** and **h**, power density distributions on the xz planes across the central axes of the SW and the FNW.

Using the 3D-FDTD simulation we obtained the FNW-SW coupling efficiencies with the side-by-side coupling scheme. Considering the nanowire's surface RMS roughness (<0.5 nm) and

the side-wall roughness peak amplitude of silicon waveguides[7] (<5 nm), the gap between the FNW and the SW was set as 5 nm. Supplementary Fig. 6 gives the calculated coupling efficiencies between an 860-nm-diameter FNW and 340-nm-height SWs with different widths (280-320 nm). As the SW width increases, we can see a red-shift of the efficiency maxima similar to Fig. 1d. Besides, the efficiencies for both coupling directions are very close around the maxima. We noted that there are small discrepancies of the efficiency dependence on SW width (shift by about 10 nm) between the simulation and the experiment, which could be attributed to errors produced in the SW-width measurement and the difference between the actual CdS refractive index and literature value[6] at telecom wavelengths.

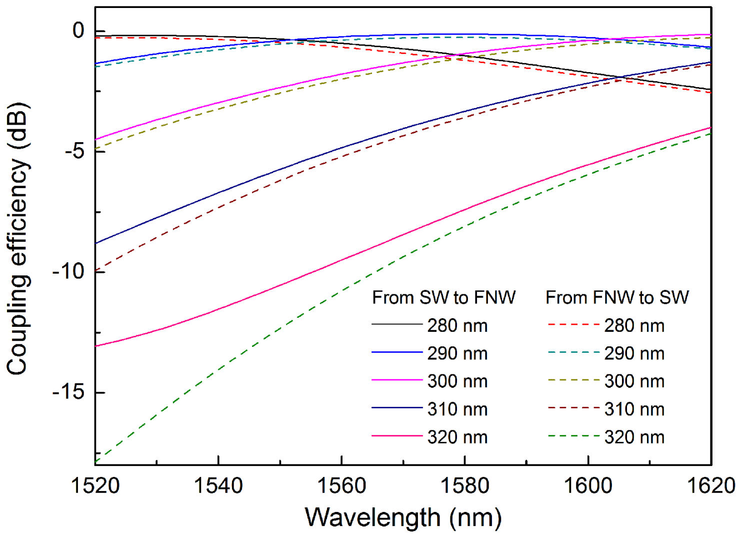

Supplementary Figure 6. Calculated coupling efficiencies between a CdS FNW and SWs. The diameter of the CdS FNW is 860 nm and the widths of the 340-nm-height SWs range from 280 nm to 320 nm. Solid lines: coupling from an SW to a FNW. Dashed lines: coupling from a FNW to an SW.

We checked the dependence of the coupling efficiency on the gap by using an FDTD simulation.

Supplementary Fig 7 gives the calculated coupling efficiencies between an 860-nm-diameter CdS FNW and a 290-nm-width SW with 3 gap sizes. As shown below, for both coupling directions, when the gap increases from 5 nm to 15 nm, the maximum efficiency decreases by about 0.3 dB and the spectrum buleshifts by about 5 nm.

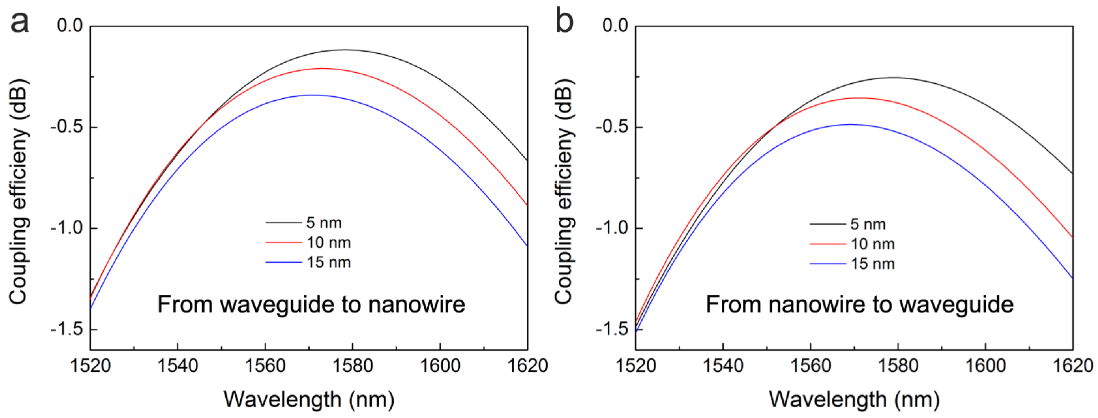

Supplementary Figure 7. Coupling efficiencies between a CdS FNW and an SW with different gaps. Calculated efficiencies for the two coupling directions, i.e., from an SW to a CdS FNW (**a**) and from a CdS FNW to an SW (**b**) with different gaps (5-15 nm). The width of the SW is 290 nm and the diameter of the CdS FNW is 860 nm.

We also calculated the coupling efficiencies between a CdS FNW and a SW in a vertical coupling scheme regarding different centre misalignments. As shown in Supplementary Fig. 8, when an 860-nm-diameter CdS FNW and a 290-nm-width SW are vertically coupled with a coupling length of 3 μm, misalignment up to 200 nm only causes 0.4-dB reduction in coupling efficiency. The robustness can be attributed to the strong evanescent field overlap of the FNW and the SW.

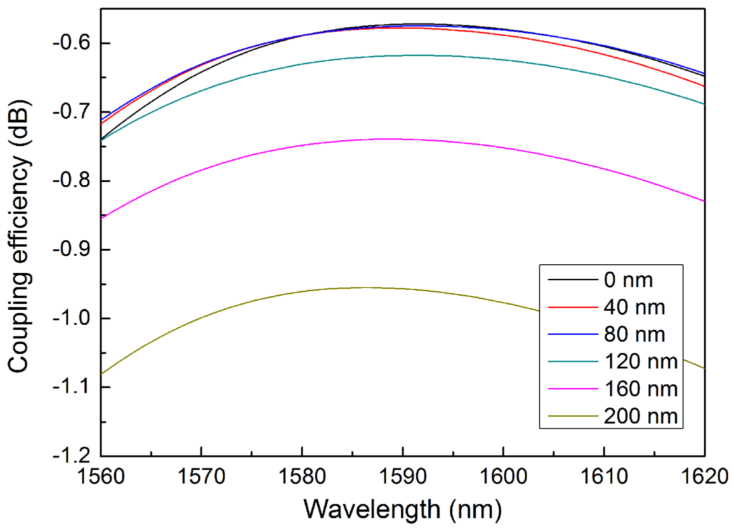

Supplementary Figure 8. Coupling efficiencies from an 860-nm-diameter CdS FNW to a 290-nm-width SW in a vertical coupling scheme with different centre misalignments

Supplementary Note 5. An experimental approach to obtaining coupling efficiency between a CdS FNW and an SW

To obtain the coupling efficiency of a CdS FNW and an SW we used a MZI-like circuit to exclude the grating-induced losses (Supplementary Figs. 9a-9c). The SW part of the circuit consists of two opposite Y branches with one arm connected by a straight SW about 26 μm in length. The splitting ratio of the Y branches is 50/50. An amplified spontaneous emission source was coupled into the input of the circuit as probing light.

First, we measured the output of the SW circuit without the CdS FNW, and denoted it as OP1 in Supplementary Fig. 9e (black triangles). Secondly, we put a graphite flake with high optical absorption on the SW to block the light propagation in the straight SW (Supplementary Fig. 9b), and obtained a much lower (-30 dB lower) output (denoted as OP2, blue circles in Supplementary Fig. 9e). Then, we bridged the open arm of the SW circuit by a CdS FNW (Supplementary Fig. 9c), and measured the output (denoted as OP3, red squares in Supplementary Fig. 9e), which goes

back to approximately the same level as the first case, indicating a high coupling efficiency between the CdS FNW and the SWs. For reference, Supplementary Fig. 9d gives an SEM image of an integrated FNW-SW MZI circuit with the bridging CdS FNW and the graphite flake. Since the input probing light was equally split into the two arms by the Y-branch, by comparing OP1 and OP3, we obtained normalized transmission of the CdS-FNW-bridged SW arm (with 2 coupling events) as OP3 - OP1, and FNW-SW coupling efficiency for single coupling event as (OP3 - OP1)/2.

In principle, this approach is valid only when the CdS FNW and the SW share a same propagation loss. When they have different losses, the actual efficiency becomes (OP3 - $loss_{\mathrm{FNW}}$ - OP1 + $loss_{\mathrm{SW}}$)/2, which means for (OP3 - OP1)/2 there is an error of ($loss_{\mathrm{SW}}$ - $loss_{\mathrm{FNW}}$)/2. However, as the loss coefficient of the SWs was 2-5 dB/cm [Ref. 8], the 26-μm-length straight SW only introduced a loss of 0.005-0.013 dB. Therefore, considering the largest loss of the SW, this error becomes (0.013 dB - $loss_{\mathrm{FNW}}$)/2, and is only 0.007 dB in maximum provided $loss_{\mathrm{FNW}}$ is negligible. On the other hand, by zooming in the spectra of the measured coupling efficiencies shown in Fig. 1d, we obtained the maximum coupling efficiency as -0.13 dB at around 1,585 nm. Considering the possible error of 0.007 dB, the actual maximum coupling efficiency should be no less than -0.14 dB (97%), which is very close to the -0.12 dB obtained from Supplementary Fig. 6.

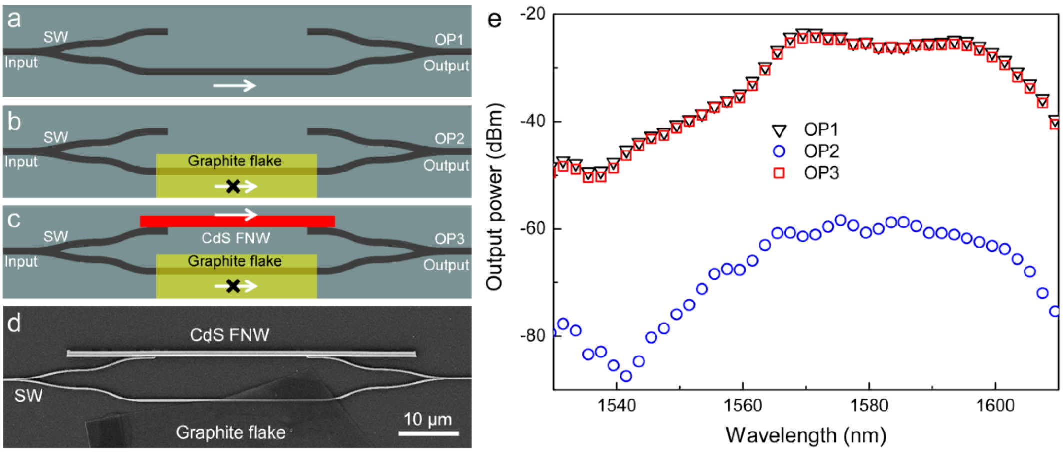

Supplementary Figure 9. Measuring the coupling efficiency of a FNW and an SW. a, Without the coupled CdS FNW, light propagates only through the straight SW. **b**, The straight SW is blocked by the graphite-induced optical loss. **c**, As the open arms of the SW circuit is bridged by the CdS FNW, light propagates through the FNW. **d**, SEM image of the integrated FNW-SW circuit with the graphite flake and the coupled CdS FNW. **e**, Measured spectra of OP1 (black), OP2 (blue), and OP3 (red) respectively.

Supplementary Note 6. Optical modulation with the hybrid MZI

In this work, we used a chopped 405-nm light (as the switch light) to modulate a CW signal light (1,574.8 nm in wavelength) propagated through the hybrid MZI by modulating optical path difference of the two arms. When being focused onto the CdS FNW/SW via a tapered fibre probe (Supplementary Figs. 10a and 10b), the 405-nm switch light generated free carriers, and thus changed refractive indices of the CdS FNW[9] / SW[10].

From the measured transmission spectrum of the MZI (red line in Supplementary Fig. 10c), we selected the wavelength of the signal light (from a tunable laser, Santec TSL-710) to be 1,574.8 nm (black arrow in Supplementary Fig. 10c), slightly shift from a valley of the oscillating transmission for higher modulation depth. Similarly, in optical modulation with the hybrid FNW-SW racetrack resonator shown in Fig. 3, the wavelength of the signal light was centred

around a resonance of the racetrack resonator, i.e., λ=1,606.2 nm. Thermal nonlinear refraction effect was believed to play a minor role here, otherwise the signs of the thermal nonlinear coefficients of CdS[11] and silicon[12] would produce opposite modulation according to the signal light position in Supplementary Fig. 10c.

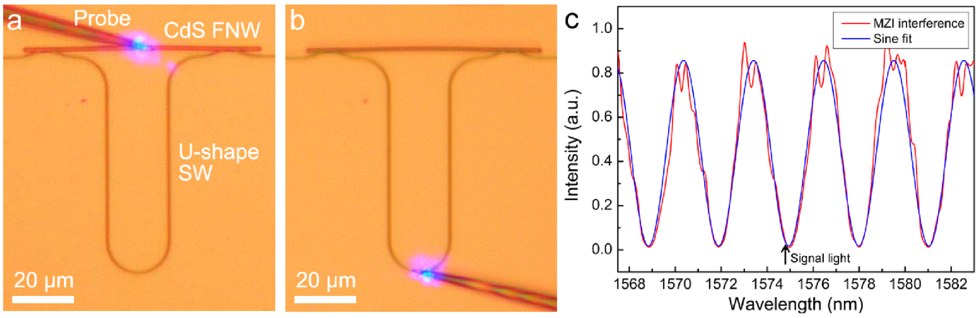

Supplementary Figure 10. a and **b**, Micrographs of illuminating the Cd FNW and the SW via a tapered fibre probe for optical modulation. **c**, Measured transmission spectrum of the MZI in linear scale with a sine fit.

For an MZI, it is important to realize 50/50 split ratios at both the input and output couplers to achieve large extinction ratios. As a supplementary investigation to the experiment, numerical calculations (Supplementary Fig. 11) for the transmissions of the couplers were also performed with the same structural parameters as in the experiment, including the bended coupling SW formed by the connected ends of two S-bends. We can see the coupling efficiencies from 1,550 nm to 1,620 nm are close to 50% and the largest split ratio is only 57/43. In addition, from the measured transmission spectrum of the MZI (Fig. 2b) high transmissions (0.46 dB in average) at the constructive interference positions were also observed as well as the large extinction ratios. These results suggest that the split ratios at both the input and output couplers were very close to 50/50.

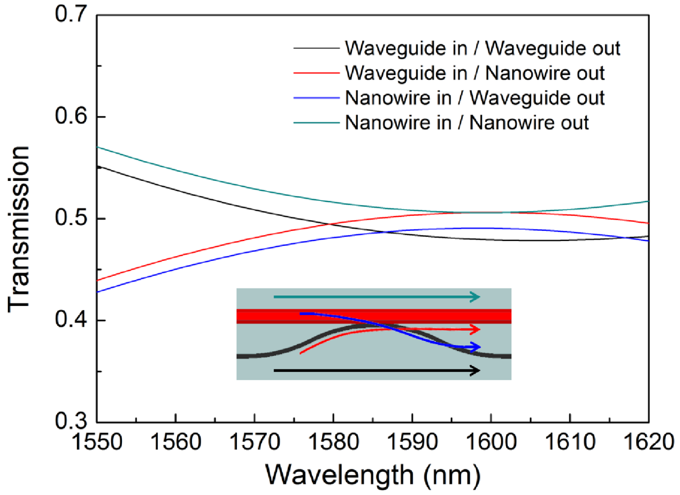

Supplementary Figure 11. Calculated transmissions from the outputs of SW and CdS FNW in the couplers used for the MZI.

When the 300-nm-diameter waveguide and 860-nm-diameter nanowire were illuminated by a Gaussian beam with a spot size of 6 μm respectively, the field intensities interacting with them were almost identical as the illuminations along their width direction were nearly uniform and the illuminated lengths were also the same. Therefore the core sizes of the waveguide and nanowire is less critical here for nonlinearity comparison.

Supplementary Note 7. Reproducibility of the integrated FNW-SW device fabrication

Before the micromanipulation with the FNWs, we used numerical simulation to determine a certain range of the structural parameters for fabrication. 20 couplers were fabricated for coupling efficiency measurement, among which there were 4 showing over 80% coupling efficiencies. For the MZIs, due to further structural optimization, half of the 20 fabricated devices showed large extinction ratio over 10 dB. As to hybrid resonators with vertical coupling scheme, the assembling was a bit more challenging because the FNW was to be suspended across two

SWs collinearly. Among the 15 hybrid resonators, there were 4 with Q factors over 1,000. For the resonators integrated with Er^{3+}/Yb^{3+}-codoped FNWs, half of the 10 devices gave maximum optical output over 200 pW. Generally, we believe better optimization in design can help reduce the difficulty in experiment.

Supplementary References

1. Hsu, Y. J., Lu, S. Y., and Lin, Y. F. One-Step Preparation of Coaxial CdS-ZnS and $Cd_{1-x}Zn_xS$-ZnS Nanowires. *Adv. Funct. Mater.* **15**, 1350-1357 (2005).
2. Guo, X., Ying, Y., and Tong, L. Photonic nanowires: From subwavelength waveguides to optical sensors. *Acc. Chem. Res.* **47**, 656-666 (2014).
3. Li, J. et al. Wavelength Tunable CdSe Nanowire Lasers Based on the Absorption-Emission-Absorption Process. *Adv. Mater.* **25**, 833–837 (2013).
4. Wu, X. et al. Hybrid Photon-Plasmon Nanowire Lasers. *Nano. Lett.* **13**, 5654–5659 (2013).
5. Li, H. H. Refractive index of silicon and germanium and its wavelength and temperature derivatives. *J. Phys. Chem. Ref. Data* **9**, 561-658 (1980).
6. Treharne, R. et al. Optical Design and Fabrication of Fully Sputtered CdTe/CdS Solar Cells. in *J. Phys.: Conf. Ser.* 012038 (2011, IOP Publishing).
7. Vlasov, Y. A. and McNab, S. J. Losses in single-mode silicon-on-insulator strip waveguides and bends. *Opt. Express* **12**, 1622–1631 (2004).
8. Chen, S. et al. Compact Dense Wavelength-Division (De)multiplexer Utilizing a Bidirectional Arrayed-Waveguide Grating Integrated With a Mach-Zehnder Interferometer*J. Lightwave Technol.* **33**, 2279–2285 (2015).

9. Li, H. P., Kam, C. H., Lam, Y. L., Ji, W. Optical nonlinearities and photo-excited carrier lifetime in CdS at 532 nm. *Opt. Commun.* **190**, 351-356 (2001).

10. Soref, R. A. and Bennett, B. R. Electrooptical effects in silicon. *IEEE J. Quant. Electron.* **23**, 123-129 (1987).

11. Venkatram, N. et al. Nonlinear absorption and scattering properties of cadmium sulphide nanocrystals with its application as a potential optical limiter. *J. Appl. Phys.* **100**, 074309 (2006).

12. Cocorullo, G., Iodice, M. and Rendina, I. All-silicon Fabry-Perot modulator based on the thermo-optic effect. *Opt. Lett.* **19**, 420–422 (1994).
